# Long noncoding RNA MALAT1 in exosomes drives regenerative function and modulates inflammation-linked networks following traumatic brain injury

**DOI:** 10.1186/s12974-018-1240-3

**Published:** 2018-07-12

**Authors:** Niketa A. Patel, Lauren Daly Moss, Jea-Young Lee, Naoki Tajiri, Sandra Acosta, Charles Hudson, Sajan Parag, Denise R. Cooper, Cesario V. Borlongan, Paula C. Bickford

**Affiliations:** 1 James A Haley Veterans Hospital, Research Service, Tampa, FL USA; 2 Department of Molecular Medicine, University of South Florida Morsani College of Medicine, 12901 Bruce B Downs Blvd, Tampa, FL 33612 USA; 3 Department of Neurosurgery and Brain Repair, University of South Florida Morsani College of Medicine, Tampa, FL USA; 4 Present address: Department of Neurophysiology & Brain Science, Graduate School of Medical Sciences & Medical School, Nagoya City University, 1 Kawasumi, Mizuho-cho, Mizuho-ku, Nagoya, Aichi 467-8601 Japan; 5 USF Health Center of Excellence for Aging and Brain Repair MDC-78, 12901 Bruce B Downs, Blvd, Tampa, FL 33612 USA

## Abstract

**Background:**

Neuroinflammation is a common therapeutic target for traumatic brain injury (TBI) due to its contribution to delayed secondary cell death and has the potential to occur for years after the initial insult. Exosomes from adipose-derived stem cells (hASCs) containing the long noncoding RNA MALAT1 are a novel, cell-free regenerative approach to long-term recovery after traumatic brain injury (TBI) that have the potential to modulate inflammation at the genomic level. The long noncoding RNA MALAT1 has been shown to be an important component of the secretome of hASCs.

**Methods:**

We isolated exosomes from hASC containing or depleted of MALAT1. The hASC-derived exosomes were then administered intravenously to rats following a mild controlled cortical impact (CCI). We followed the rats with behavior, in vivo imaging, histology, and RNA sequencing (RNA Seq).

**Results:**

Using in vivo imaging, we show that exosomes migrate into the spleen within 1 h following administration and enter the brain several hours later following TBI. Significant recovery of function on motor behavior as well as a reduction in cortical brain injury was observed after TBI in rats treated with exosomes. Treatment with either exosomes depleted of MALAT1 or conditioned media depleted of exosomes showed limited regenerative effects, demonstrating the importance of MALAT1 in exosome-mediated recovery. Analysis of the brain and spleen transcriptome using RNA Seq showed MALAT1-dependent modulation of inflammation-related pathways, cell cycle, cell death, and regenerative molecular pathways. Importantly, our data demonstrates that MALAT1 regulates expression of other noncoding RNAs including snoRNAs.

**Conclusion:**

We demonstrate that MALAT1 in hASC-derived exosomes modulates multiple therapeutic targets, including inflammation, and has tremendous therapeutic potential for treatment of TBI.

## Background

Approximately two million Americans suffer from traumatic brain injury (TBI) annually, accounting for 30% of all injury-related fatalities [1, 2]. Currently, treatment options for TBI consist predominantly of rehabilitation and symptom management. Thus, there is an urgent need for novel treatments to prevent or slow the progression of secondary injury in TBI. Due to its contribution to secondary cell death, which can occur years after the initial insult, neuroinflammation is a common target for prospective TBI therapeutics [3–8]. Recruitment of macrophages into the area of brain damage is an important aspect of secondary injury, and numerous approaches have been explored to intervene in this process [9–12]. One of the organs important in the flux of monocytes and T cells into the peripheral circulation following injury is the spleen, and it has been demonstrated that the intact spleen is important for the neuroprotective action of multipotent adult progenitor cells after several models of brain insult [13–16].

The neuroprotective capacity of mesenchymal stem cells (MSCs) derived from adipose tissue has garnered great interest in regenerative medicine. Human adipose-derived stem cells (hASCs) manifest a secretome that is capable of modulating the environment of the host. Among these secreted molecules, small membrane-bound vesicles known as exosomes have gained particular interest due to their diverse cargo including cellular proteins, mRNA, and noncoding RNAs, as well as their ability to evade immune rejection by the host and modify immune responses [17]. Compelling evidence suggests that exosomes play an important role in cell-to-cell communication, and rats or mice treated with exosomes have demonstrated improved functional recovery after models of stroke and TBI [18–20]. These MSC-derived extracellular vesicles are also thought to modulate immune function as part of their mechanism of prevention of injury following TBI [21].

Long noncoding RNA (lncRNA) are important regulators of gene expression and have been shown to mediate several pathways such as cell cycle, proliferation as well as apoptosis, and immune modulation. Additionally, they are involved in epigenetic regulation, transcription, and translation as well as alternative splicing of genes. Aberrant expression of lncRNAs is associated with human diseases [22–25]. Of the cargo secreted in exosomes, lncRNA metastasis-associated lung adenocarcinoma transcript 1 (MALAT1) controls key biological processes such as cellular proliferation and differentiation and is thought to have a special role in regeneration after injury [26–28]. We previously used the secretome (collected as conditioned media) of hASC as treatment and showed that it promotes repair following TBI [26]. MALAT1 is part of the cargo secreted by hASC [29], and our in vitro studies using the HT22 cell line demonstrated that MALAT1 promotes survival and proliferation [27]. Here, we sought to examine the use of hASC-derived exosomes as a novel, cell-free regenerative approach to injury recovery in an in vivo model of TBI. We identify the cellular processes crucial to recovery that are specifically mediated by MALAT1. Importantly, we also elucidate the genomic impact exosome treatment using RNA sequencing (RNA Seq) transcriptome analysis. Our results show that MALAT1 in hASC-derived exosomes is therapeutically beneficial after TBI.

## Methods

### Exosome isolation and collection from ASCs

hASC (Zenbio, catalog #ASC-F) were trypsinized, and cell pellets were collected in 100 μL Nucleofector® kit (Lonza, catalog #VPE-1001) and combined with pMAX GFP (2 μg). The cell/DNA solution was transferred to a cuvette, and the program initiated (0.34 kV, 960 μF). Medium (500 μL) was added immediately, and cells were gently transferred to 100-mm plates and allowed to grow for 48 h. To deplete MALAT from hASC exosomes, we used MALAT1 antisense oligonucleotide (ASO; ID 39524 ASO from Ionis Pharmaceuticals), validated for specificity and designed for efficient uptake by cells. The ASOs were added to hASC and incubated for 48 h. The expression levels of MALAT1 were verified in the exosomes using human MALAT1 primers in qPCR. Exosomes were isolated from conditioned media (CM) and verified that exosomes contain GFP as previously described by our lab [27, 29]. Briefly, CM derived from hASC was collected after 48 h and centrifuged at 3000*g* for 15 min to remove dead cells. ExoQuick™ (System Biosciences, Catalog #EXOTC-50A-1) reagent was added to the CM and incubated overnight at 4 °C along with XenoLight 1,1-dioctadecyl-3,3,3,3-tetramethylindotricbocyanine iodide (DiR) (catalog #125964; Caliper Life Sciences). Following centrifugation at 1500*g* for 30 min, the pellet was further processed. ExoCap™ (JSR Life Sciences, Catalog #EX-COM) composite reagent containing magnetic beads for CD9, CD63, and CD81 was used to purify exosomes. Exosomes were eluted from beads using 500 μL of the manufacturer’s elution buffer. Buffer was then exchanged by Ambicon columns. Nanoparticle tracking analysis from NanoSight (NTA3.1, Build 3.1.46 RRID SCR-014239) was used to analyze peak diameter and concentration of exosomes obtained from 10^6^ hASC. Analysis showed exosome size to be 89 ± 7 nm.

### Animal model and surgical procedures

The University of South Florida Institutional Animal Care and Use Committee (IACUC) approved all experimental procedures with animals. All rats were housed under normal conditions (20 °C, 50% relative humidity, and a 12-h light/dark cycle). All studies were performed by personnel blinded to the treatment condition.

A total of 79 Fisher 344 male rats were subjected to either TBI by controlled cortical impact or sham surgery. The rats were randomly distributed into the following groups: surgery with no TBI (sham control C, *N* = 11), TBI with unconditioned media as vehicle (T; *N* = 20), TBI treated with exosomes (TE, *N* = 18), TBI treated with exosomes depleted of MALAT1 (TEdM, *N* = 20), and TBI with injection of conditioned media depleted of exosomes (TdCM; *N* = 7). Deep anesthesia was administered to all rats undergoing TBI surgery using 1–2% isoflurane in nitrous oxide/oxygen (69%/30%) and was maintained using a gas mask. TBI-induced animals were fixed in a stereotaxic frame (David Kopf Instruments). The TBI procedure was performed as previously described [26]. Briefly, the skull was exposed by a midline incision, coordinates of + 0.2 mm anterior and + 0.2 mm lateral to the midline were found, and craniotomy was performed; the brain was then impacted at the frontoparietal cortex with a velocity of 6.0 m/s reaching a depth of 0.5 mm (mild TBI) below the dura matter layer and a dwell time of 150 ms. Body temperature of the animals was maintained within normal limits by a computer-operated thermal pad and rectal thermometer. All animals were closely monitored postoperatively and analgesic ketoprofen was administered prior to surgery and as needed thereafter. Rats were maintained on regular rodent diet throughout the experiment.

### Intravenous administration of exosomes and CM

Three hours after TBI induction, rats were anesthetized using 1–2% isoflurane in nitrous oxide/oxygen (69%/30%) and were maintained using a gas mask. Intravenous injections through the jugular vein were then performed and divided as follows: TBI-Veh (T) received 500-μl unconditioned media, TBI animals with exosomes depleted conditioned media (TdCM) received 500-μl conditioned media depleted of exosomes, TBI animals with exosomes (TE) received exosomes (100 μg in 500 μl of sterile saline), and TBI animals with exosomes depleted of MALAT1 (TEdM) received exosomes (100 μg in 500 μl of sterile saline). Sham animals did not receive any injection, and animals receiving unconditioned media were used as control groups. To evaluate the migration of the transplanted exosomes, exosomes were incubated with DiR during isolation.

### XenoLight DiR for in vivo and ex vivo biodistribution imaging procedures

To visualize DiR fluorescence emitted from the injected exosomes in vivo, animal’s abdomens were shaved to avoid light scattering and anesthetized in a chamber with 3.0% isoflurane. Animals were then transferred from the chamber to the IVIS Spectrum 200 Imaging System (Xenogen), and the isoflurane level was maintained at 1–2% throughout image acquisition. The biodistribution of DiR-labeled exosomes were monitored in vivo at 1, 4, 12, 24, 48, and 72 h and again at 11 days post-surgery and transplantation. Images were also obtained of ex vivo organs including the brain, liver, lungs, and spleen after 1, 3, 7, and 11 days post-surgery and transplantation. Full-body imaging was performed ventrally at all time points, and a second set of images were obtained for the head region using a higher magnification. The parameters used throughout the experiment were as follows: exposure time = auto; lamp voltage = high; F/stop = 2; binning = 8; excitation filter = 745 nm; emission filter = 800 nm; field of view = D (for whole body), C (for ex vivo organs), or B (for head region). All captured images were analyzed using Living Image Software 4.0 (Xenogen RRID:SCR_014247). To analyze the change in DiR fluorescence intensity, identical regions of interest (ROIs) were placed on the abdomen and head area for all animals. The same ROI was also placed on the control animal as the background reference. Background efficiency was subtracted from each of the individual animal’s efficiency and presented as an average radiant efficiency (photons per second per square centimeter per steridian divided by microwatts per square centimeter).

### Behavioral testing

Each rat was subjected to a series of behavioral tests to assess motor and neurological performance of animals, at baseline before surgery and post TBI at days 0, 3, and 7.

#### Elevated body swing test (EBST)

EBST is a measure of asymmetrical motor behavior that does not require animal training or drug injection [30]. The rats were held, in the vertical axis, 1 in. from the base of its tail and then elevated to an inch above the surface on which it has been resting. The frequency and direction of the swing behavior were recorded for 20 tail elevations. A swing was counted when the head of the rat moved 10° from the vertical axis to either side. A score of 10 indicates no bias. The higher number of swings made to one side was added per group and divided by the *n*, giving us the average number of biased swings per treatment group.

#### Forelimb akinesia

Before and after TBI surgery, rats from all groups were evaluated for forelimb akinesia [31]. Ipsilateral and contralateral forepaw strength and motility were scored by two experimentally blinded evaluators using the following forelimb akinesia scale. On a scale of 1 to 3, 1 is normal, 2 is impaired, and 3 is severely impaired. Scores were tallied for each individual animal, and then, mean scores for treatment groups were used for analyses.

#### Paw grasp

Before and after TBI surgery, grip strength of rats from all groups was evaluated. An abnormal grip is indicative of impaired neuromuscular function. In this test, rats were held by their bodies against a pole. Both ipsilateral and contralateral paw grip strength were scored by two experimentally masked evaluators using the following grip strength scale. On a scale of 1 to 3, 1 is normal, 2 is impaired, and 3 is severely impaired. Scores were tallied for each individual animal, and then, mean scores for treatment groups were used for analyses.

### Brain and organ harvesting, fixation, and sectioning

Under deep anesthesia, rats were sacrificed on days 1, 3, 7, and 11 following TBI for protein analysis, RNA sequencing, and/or immunohistochemical investigations. Briefly, animals were perfused through the ascending aorta with 200 ml of cold PBS, followed by 200 ml of 4% paraformaldehyde in phosphate buffer (PB). The brains, spleen, lungs, and liver were removed and post-fixed in the same fixative for 24 h, followed by 30% sucrose in PB until completely sunk. Series of coronal sections were cut at a thickness of 40 um with a cryostat and stored at − 20 °C. Six coronal sections between the anterior edge and posterior edge of the impacted area were collected and processed for GFP expression in exosome-injected animals.

### Measurement of impact and peri-impact area: Nissl staining analysis

**Fig. 1:**
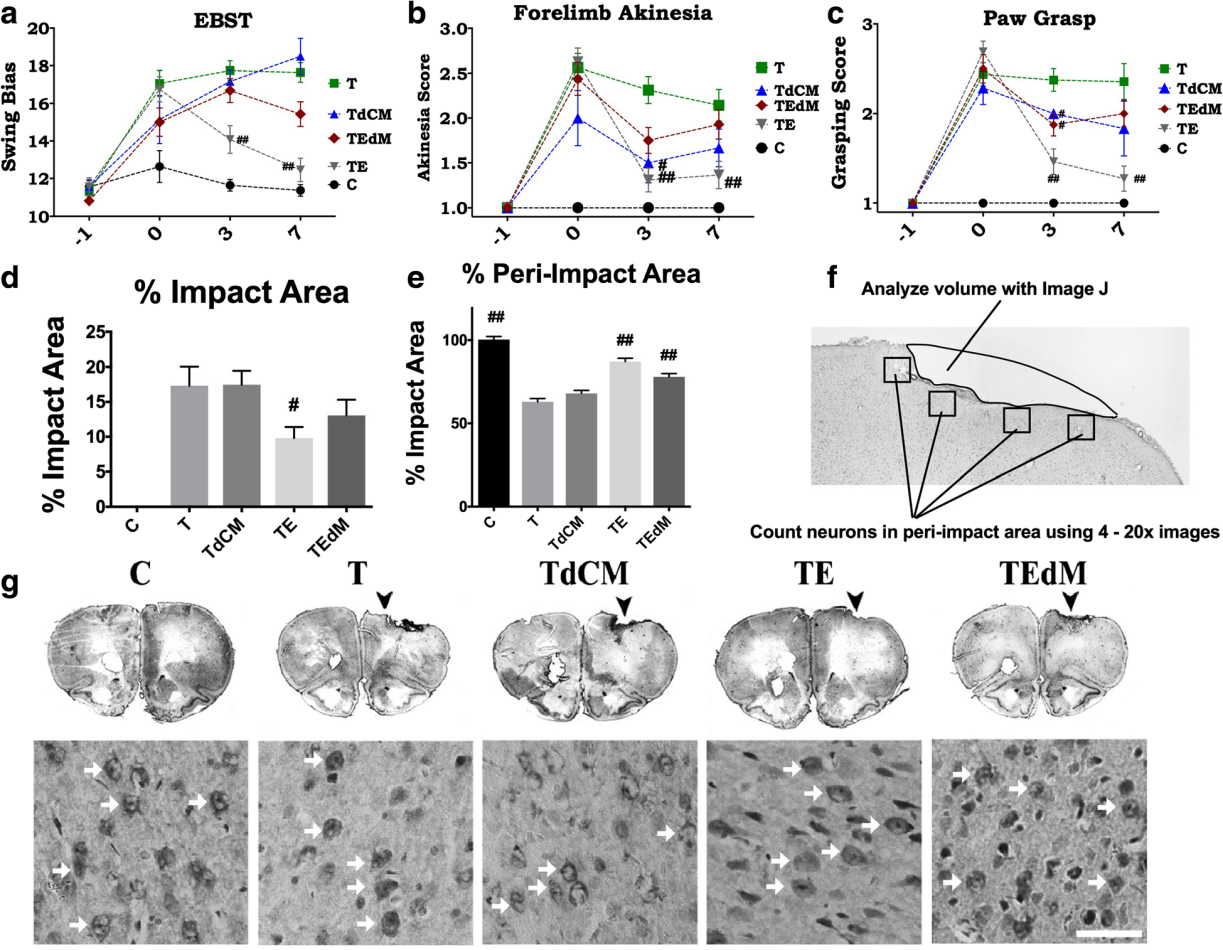
Treatment with exosomes (TE) significantly rescued the TBI-associated motor deficits relative to TBI-Veh (T) and controls (C). Graphs show motor assessment using EBST (**a**), forelimb akinesia (**b**), and paw grasp test (**c**). Two-way ANOVA showed significant effects as follows: EBST, treatment effects *F*(4) = 27.04; forelimb akinesia treatment effect *F*(4) = 30.3; paw grasp treatment effect *F*(4) = 42.2. Post hoc Bonferroni multiple comparisons are reported for differences versus TBI vehicle (T). ^#^*p* < 0.01, ^##^*p* < 0.001. Treatment with exosomes depleted of MALAT1 (TEdM) did not improve motor performance on EBST and only improved forelimb akinesia and paw grasp at day 3. Treatment with conditioned media depleted of exosomes (TdCM) also showed no improvement on EBST and only improved scores at day 3 on the other two tasks. Groups: sham *N* = 11; TBI vehicle *N* = 16; TBI exosomes *N* = 16; TBI exosomes depleted of MALAT1 *N* = 16; TBI-conditioned media depleted of exosomes *N* = 7. Lesion assessment: treatment with exosomes derived from hASCs significantly reduces impact and peri-impact areas of rats after mild TBI. Nissl staining as shown in **f** was performed on day 11 to assess damage to cortical region post TBI. Graphs **d** and **e** quantify the data from images. **f** The methods for quantifying the impact area and for choosing images for analysis of the peri-impact area. Data for impact area (**d**) showed significant reduction in cortical lesion area following treatment with exosomes in the TE group and no rescue by any other treatment. Representative images of sections used for quantifying impact area and peri-impact are shown (**g**). For the peri-impact area (**e**), there was a significant rescue in the TE group, whereas TEdM group displayed partial rescue of the peri-impact areas when compared with vehicle (T) and sham controls (C). Data in the bar graphs represent the mean ± SEM values. Impact area *F* = 14.78; peri-impact area *F* = 56.58. Data were analyzed by one-way ANOVA followed by Dunnet’s multiple comparison test. #*p* < 0.1, ^##^*p* < 0.01

Serial sections corresponding to the same group of animals were stained with Nissl (Thermo Fisher Scientific Cat# N21483 RRID:AB_2572212) for impact and peri-impact calculations. Six coronal sections between the anterior edge and posterior edge of the impacted area were collected and processed for Nissl staining from each brain perfused at day 11 after TBI. Sections were cut at a thickness of 40 μm by a cryostat. Coronal tissue sections were randomly selected for measurement of impact and peri-impact area. Brain sections were examined using a light microscope (Olympus) and Keyence microscope. The impact area was outlined and measured in each of the six slices and quantified by a computer-assisted image analysis system (NIH Image RRID:SCR_003070). To measure the peri-impact area, we examined the same six sections through the impact zone and placed four 20× fields along the edge of the impact core. Each 20× image was then analyzed for the number of live neurons with Nissl staining (see Fig. 1f for example). Impact and peri-impact area was then expressed as a percentage of the ipsilateral hemisphere compared with the contralateral hemisphere.

#### RNA sequencing: RNA quality control

RNA was isolated from the brains (area near impact site) and spleens of rats at day 7 following TBI in the sham surgery—control (C), TBI with vehicle (T), TBI treated with exosomes (TE), and TBI treated with MALAT1-depleted exosomes (TEdM) groups. Four rats from each group were randomly chosen and pooled to maximize biological diversity and sent for RNA Seq (Ocean Ridge Biosciences). Eight total RNA samples (four rat spleen tissue and four rat brain tissue) were submitted to Ocean Ridge Biosciences for RNA sequencing. RNA was quantified by O.D. measurement and assessed for quality on a 1% agarose–2% formaldehyde RNA quality control (QC) gel. The RNA was then digested with RNase-free DNase I (Epicentre; Part # D9905K) and re-purified on RNeasy MinElute columns (Qiagen; Part # 74204) using an alternative high ethanol protocol to preserve low molecular weight (LMW) RNAs. The newly purified RNA samples were then quantified by O.D. measurement.

#### Library preparation

Ribosomal RNA was depleted from 1 μg of DNA-free total RNA using the Ribo-Zero Gold rRNA Removal Kit for Human/Mouse/Rat (Illumina, part number MRZG126). Template DNA molecules suitable for cluster generation were then prepared from the rRNA-depleted samples using the ScriptSeq V2 RNA-Seq Library Prep Kit (Illumina, part number SSV21124). The quality and size distribution of the amplified libraries were determined by chip-based capillary electrophoresis on an Agilent 2100 Bioanalyzer. Libraries were quantified using the KAPA Library Quantification Kit (Kapa Biosystems, Boston, MA).

#### Sequencing

The libraries were pooled at equimolar concentrations and diluted prior to loading onto the flow cell of the Illumina cBot cluster station. The libraries were extended and bridge amplified to create sequence clusters using the Illumina HiSeq PE Cluster Kit v4 and sequenced on an Illumina HiSeq Flow Cell v4 with 50-bp paired-end reads plus index read using the Illumina HiSeq SBS Kit v4. Real-time image analysis and base calling were performed on the instrument using the HiSeq Sequencing Control Software version 2.2.58. All samples had a minimum of 43,303,826 passed-filter single-end reads. The sequences aligned at an average of 72% ± 2% (SD) efficiency to the reference genome.

#### Alignment to genome

Sequence alignment was performed using TopHat v1.4.1 (RRID SCR-013035) with the following settings:

tophat -p 2 -o [OUTPUT FOLDER] --library-type fr-secondstrand –r [Dx] –mate-std-dev [Ds] [rn5 BOWTIE GENOME INDEX] [FASTQ 1] [FATSQ 2]

where Dx and Ds represent sample-specific values for the mean and standard deviation, respectively, of the inner distance between reads, as determined by non-gapped alignment to rat mRNA. rn5 BOWTIE GENOME INDEX was built from the soft-masked genome sequence. In practice FASTQ files for each sample were split into multiple FASTQs having 4 million reads each in order to accelerate processing.

#### ncRNA counting and annotation

The number of reads aligning to each ncRNA feature were counted using BEDtools v2.16.2 (RRID SCR-006646) with the following settings: bedtools multicov -bams C80R4ANXX_s8illumina12index_4_SL134409.fastq_05.bam -bed rnacentral_active_v3_rat-mouse-human.gff C80R4ANXX_s8illumina12index_4_SL134409.fastq_05/counts.txt.

In practice, FASTQ files for each sample were split into multiple FASTQs having 4 million reads each in order to accelerate processing. For each sample, the resulting BAM files were merged as well as the count files. For all ncRNAs having at least one read aligned, annotations were added using RNAcentral’s RESTful API. The RPKM values were filtered to retain a list of ncRNAs with an RPKM equivalent to 50 mapped reads in one or more samples. The threshold of 50 mapped reads per ncRNA is considered the Reliable Quantification Threshold, since the RPKM values for a ncRNA represented by 50 reads should be reproducible in technical replicates. To avoid reporting large fold changes due to random variation of counts from low abundance ncRNA, RPKM values equivalent to a count of <= 10 reads per ncRNA were replaced in the following way. First, for each sample, the RPKM value equivalent to 10 reads/ncRNA was calculated (assuming a median ncRNA length of 0.122 kb). These RPKMs were then averaged across all the samples in the experiment, and this average value was used for replacement.

#### Fold change calculations

The filtered RPKM data for 19,058 detectable rat genes (RPKM values > Reliable Quantification Threshold (RQT) in at least one sample) were used to calculate the fold changes for TBI exosome/TBI vehicle, TBI exosome MALAT1/TBI exosome, and TBI vehicle/no TBI independently for the spleen and brain samples. Fold changes are expressed as the negative reciprocal in tables and for additional analysis. The splicing index was calculated based on the following formula: exon RPKM/gene RPKM.

#### Hierarchical clustering

Similarly, the same RPKM data for all 19,058 detectable rat genes were used for hierarchical clustering analysis by Cluster 3.0 software^4^ (RRID SCR-013505). Genes were log-2 transformed and median centered prior to hierarchical clustering. Hierarchical clustering was conducted on genes and samples using centered correlation as the similarity metric and average linkage as the clustering method. The intensity scale shown is arbitrary.

#### Real-time qPCR using SYBR green

Total RNA as described above for RNA Seq was used for validation. One microliter of cDNA was amplified by real-time quantitative PCR using Maxima SYBR Green/Rox qPCR master mix (Thermo Scientific) in an ABI ViiA7 sequence detection system (PE Applied Biosystems) to quantify the relative levels of the transcripts in the samples. The primers were GAPDH sense primer 5′- TGACGTGCCGCCTGGAGAAAC -3′ and antisense 5′- CCGGCATCGAAGGTGGAAGAG -3′; human MALAT1 sense 5′ CTTCCCTAGGGGATTTCAGG 3′ and antisense 5′ GCCCACAGGAACAAGTCCTA 3′; rat MALAT1 sense 5’ TGCAGTGTGCCAATGTTTCG 3′ and antisense 5′ GGCCAGCTGCAAACATTCAA 3′; U1snRNA sense 5′ TCCCAGGGCGAGGCTTATCCATT 3′ and antisense 5′ GAACGCAGTCCCCCACTACCACAAAT 3′.

Real-time PCR was then performed in triplicate on samples. The plate setup included a no template control, no RNA control, no reverse transcriptase control, and no amplification control. After primer concentrations were optimized to give the desired standard curve and a single melt curve, relative quotient (RQ) was determined using the ∆∆C_T_ method with U6snRNA or GAPDH as the endogenous control and no TBI sample as the calibrator sample. Experiments were repeated four times.

For absolute quantification, a standard curve was generated for each gene in every assay. Briefly, 100 to 0.4 ng of RNA were reverse transcribed as described above. The resulting cDNA was used to obtain a standard curve correlating the amounts with the threshold cycle number (*C*_t_ values). A linear relationship (*r*^2^ > 0.96) was obtained for each gene. Real-time PCR was then performed on samples and standards in triplicates. The plate setup also included a standard series, no template control, no RNA control, no reverse transcriptase control, and no amplification control. The dissociation curve was analyzed for each sample. Absolute quantification of mRNA expression levels for MALAT1 or GFP was calculated by normalizing the values to GAPDH.

## Results

### Exosome treatment improves motor impairment and reduces lesion volume in a MALAT1-dependent manner

Fisher 344 male rats were subjected to either TBI by controlled cortical impact (CCI) injury model or no TBI (sham surgery only, control group) and treated with hASC-derived exosomes 3 h after surgery. Surgery is described in the “Methods” section. The rats were randomly distributed into the following groups: surgery with no TBI (sham control C, *N* = 11), TBI with unconditioned media as vehicle (T; *N* = 20), TBI treated with exosomes (TE, *N* = 18), TBI treated with exosomes depleted of MALAT1 (TEdM, *N* = 20), and TBI with injection of conditioned media depleted of exosomes (TdCM). TdCM-treated animals were included to delineate the effect of the exosomes from other biomolecules of the secretome and because our previous study had demonstrated that conditioned media harvested from hASCs was an effective treatment post TBI [26]. Each rat was subjected to a series of behavioral tests including elevated body swing test (EBST), forelimb akinesia, and paw grasp to reveal motor and neurological performance of animals, before injury and after TBI on days 0, 1, 3, and 7.

The EBST records the number of lateral swings to one side or the other and defines a swing bias to one side as a motor defect. A score of 10 equals no bias, and higher scores indicate asymmetry. EBST revealed that rats subjected to TBI exhibited heightened swing bias and therefore significant asymmetry in motor activity after injury. TBI (T) animals displayed no recovery when tested on days 3 and 7, whereas significant recovery of motor symmetry was detected in TBI exosome-treated rats (TE) (two-way ANOVA followed by Tukey’s post hoc, *p* < 0.01 versus TBI-Veh; Fig. 1a). Treatment with either conditioned media depleted of exosomes (TdCM) or exosomes depleted of MALAT1 (TEdM) did not affect recovery of the animals, and scores for these groups were not significantly different from the TBI-vehicle (T) group).

The forelimb akinesia test indicated an apparent loss of motor strength in TBI rats after injury when compared with sham controls. TBI-Veh (T) animals retained impairment through day 7 and did not display any significant amelioration of forelimb akinesia. TBI animals treated with exosomes (TE) recovered by day 3 (Fig. 1b), whereas TBI animals treated with conditioned media depleted of exosomes (TdCM) had only modest recovery after day 3, but not day 7.

Assessment of paw grasp function is indicative of TBI-associated loss of strength and was apparent in all animals subjected to injury. After day 3 post TBI, TBI exosome-treated (TE) animals scored significantly better on the paw grasp test compared to TBI-vehicle-treated (T) animals. The animals in the conditioned media depleted of exosomes and the exosomes depleted of MALAT1 groups again showed a modest improvement at day 3 that was not maintained at day 7 (Fig. 1c). Results show only the cohort treated with exosomes maintained this significant treatment effect on the paw grasp assessment demonstrating continued recovery of strength for the exosome-treated group compared to TBI sham treatment (two-way ANOVA followed by Tukey’s post hoc, *p* < 0.001).

We performed Nissl staining to quantify the lesion volumes and surrounding damage after TBI in the peri-impact area. Administration of exosomes was found to significantly reduce both the impact (Fig. 1d) and peri-impact (Fig. 1e) area of TBI-associated injury in the ipsilateral hemisphere. Exosomes depleted of MALAT1 (TEdM) had moderate improvement in peri-impact measures, but not in impact area (Fig. 1d, e). Treatment with conditioned media depleted of exosomes did not show recovery in either the impact or the peri-impact area after TBI injury. Examples of images used for analysis are shown in Fig. 1g, as well as examples of images used to quantify the live neurons in the peri-impact area.

### Imaging of exosomes in vivo shows distribution primarily to the abdomen and head regions

**Fig. 2:**
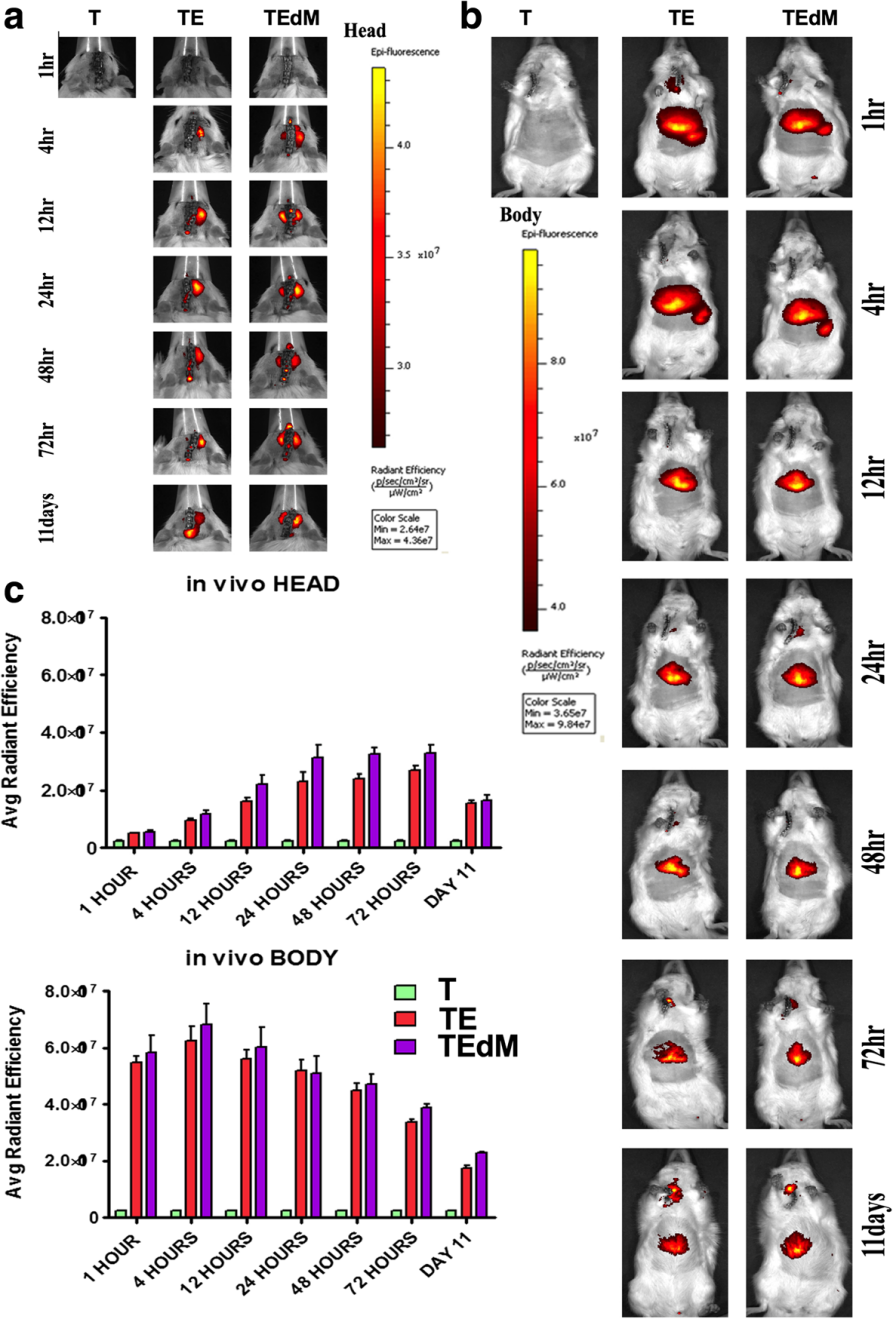
In vivo biodistribution of DiR-labeled exosomes after TBI (**a**, **b**). Imaging revealed exosomes migrated robustly to the spleen and liver at 1 and 4 h after transplantation before gradually decreasing. Exosomes also migrated to the impact site of the brain 4 h after transplantation and steadily increased through 72 h relative to sham controls. Graphs in **c** show radiant efficiency is expressed as photons per second per square centimeter per steridian divided by microwatts per square centimeter(((*p*/*s*)/ 〖 *cm*) ^ 2/*sr*)/(*μW*/ 〖 *cm*) ^ 2 )). Data in bargraphs represent the mean ± SEM values *N* = 3 per group

We determined the biodistribution of XenoLight DiR-labeled exosomes in vivo in rats with CCI treated with exosomes and exosomes depleted of MALAT1. Exosomes were administered through the jugular vein of TBI animals 3 h after injury, and their biodistribution was tracked at time points 1, 4, 12, 24, 48, and 72 h and 11 days after surgery (Fig. 2). Minimal DiR signal was detected in both the head and body regions of the sham control animals as expected which provided a reference value throughout the analysis. Within the first hour of transplantation, no noticeable signal was observed in the head region of either treatment groups, whereas a high level of fluorescent signal was present in the abdomen of both TBI groups treated with either exosomes (TE) or exosomes deleted of MALAT1 (TEdM) animals. Fluorescent signal slightly increased after 4 h for both groups in both the head and abdomen regions of the animals. At the 12-h time point, the signal in the head region had increased from the previous time points and continued to increase at 24 h where it seemed to plateau through 48 and 72 h until decreasing on day 11 after transplantation. The abdomen region instead displayed the opposite trend and showed a gradually decreasing DiR signal after 4 h that continued to decline at the 12-, 24-, 48-, and 72-h time points with the lowest radiant efficiency observed on day 11. Quantitative analysis confirmed these observations, as shown in Fig. 2a, b. Throughout all the data points, the group treated with exosomes depleted of MALAT1 showed a slightly higher average radiant efficiency while following the same trend as exosome-treated animals, but this did not achieve significance.

### Imaging of exosomes ex vivo in organs shows localization primarily to the spleen and brain

**Fig. 3:**
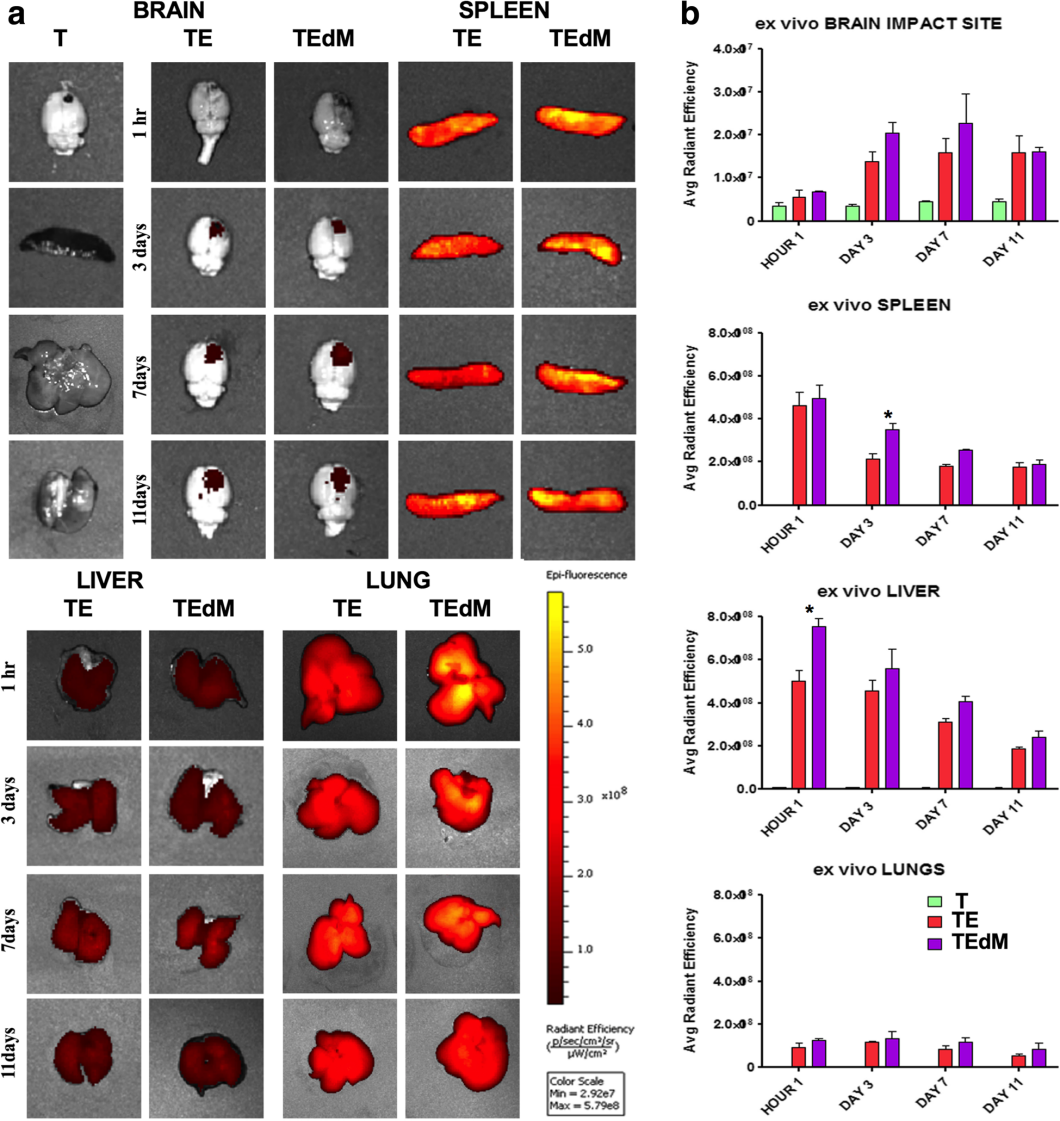
Ex vivo biodistribution of DiR-labeled exosomes after TBI. **a** Imaging revealed exosomes migrating to the spleen, liver, and lungs 1 h after transplantation and migrating to the impact site of the brain on day 3 after TBI relative to sham controls. Ex vivo analysis shows the fluorescent signal intensity was highest in the spleen and liver 1 h after transplantation and gradually declined through day 11, whereas the lungs slightly increased at day 3 before declining through days 7 and 11. The brain impact site showed no apparent signal when compared to TBI-sham treatment animals 1 h after exosome transplantation, but increased by day 3 where the signal remained through day 7 and only TEdM radiant efficiency declined by day 11. **b** Radiant efficiency is expressed as photons per second per square centimeter per steridian divided by microwatts per square centimeter(((*p*/*s*)/ 〖 *cm*) ^ 2/*sr*)/(*μW*/ 〖 *cm*) ^ 2 )). Data represent the mean ± SEM values *N* = 3 per group

Next, we determined the biodistribution of XenoLight DiR-labeled exosomes ex vivo in organs of rats with CCI treated with exosomes and exosomes depleted of MALAT1 when administered as described above for in vivo imaging. A separate cohort was examined, and each organ including the brain, lungs, liver, and spleen was imaged ex vivo at 1 h and at 3, 7, and 11 days following TBI (Fig. 3a–e). One hour after administration, the highest signal was observed in the spleen whereas the liver and the lungs showed lower signal intensity and there was no signal observed in the brain. Interestingly, there was higher signal for the exosomes depleted of MALAT1 (TEdM) in the liver at 1 h suggesting a higher rate of clearance initially. At day 3, a reduction in signal was observed in the spleen and liver and this continued over time. Signal intensity in the brain, however, increased on day 3 and continued to increase through day 7.

### RNA sequencing transcriptome analysis identifies cellular processes crucial to recovery that are specifically mediated by MALAT1

To gain insight into genomic changes following CCI and its treatment with hASC-derived exosomes, we performed RNA sequencing. Transcriptome analysis provides valuable knowledge of genomic changes following brain injury and the response to treatment with exosomes. We isolated RNA from the brains, specifically an area near the impact site, and the spleens of rats at day 7 following TBI in the sham surgery—control (C), TBI with vehicle (T), TBI treated with exosomes (TE), and TBI treated with MALAT1-depleted exosomes (TEdM) groups. Four rats of each group were randomly chosen and pooled to maximize biological diversity and sent for RNA Seq (Ocean Ridge Biosciences). The percent of mapped reads to total genome over total read across all sequencing was 96 ± 0.7%.

**Fig. 4:**
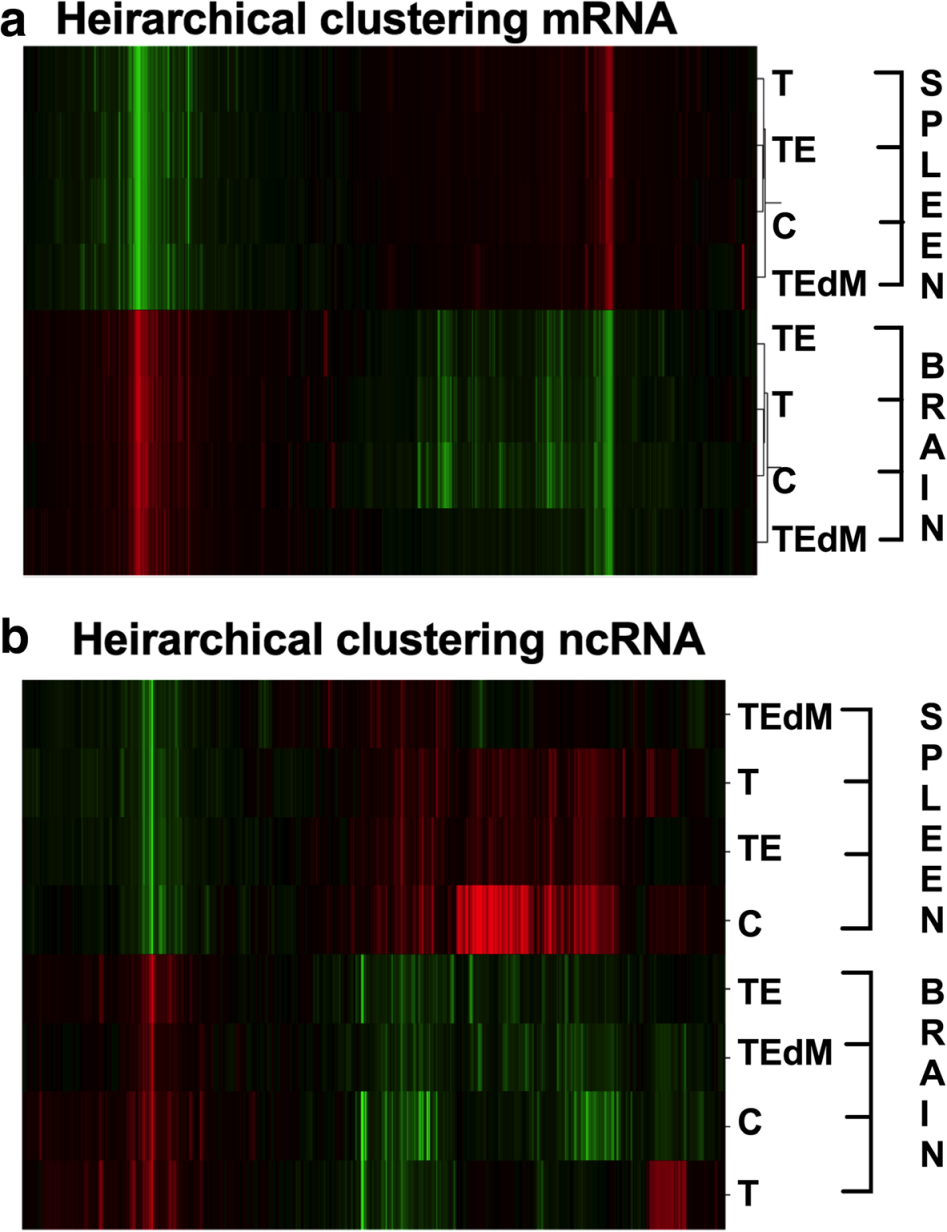
Hierarchical heat map of gene expression of (**a**) coding genes or (**b**) noncoding RNA from the RNA Seq dataset. The gene clustering from the spleen is on the top, and brain is the lower half of the dendrograms. For both **a** and **b**, RPKM data from 19,058 detectable rat genes were used in analysis by hierarchical clustering with Gene Cluster 3.0. RPKMs were log-2-transformed and adjusted by centering genes using the median. Clustering was performed on genes and arrays using centered correlation as distance measure and average linkage as method. Data shows clusters of genes or noncoding RNA that are affected by TBI and restored towards the no TBI pattern with exosome treatment

We performed hierarchical clustering of the entire RNA Seq transcriptome data in order to evaluate the relationship between gene expression profiles in the brain or spleen with respect to the different treatments, i.e., no TBI sham surgery control (C), TBI treated with vehicle (T), TBI treated with exosomes (TE), and TBI treated with exosomes depleted of MALAT1 (TEdM). The same RPKM data for all 19,058 detectable genes were used for hierarchical clustering analysis by Cluster 3.0 software. Genes were log-2 transformed and median centered prior to hierarchical clustering. Hierarchical clustering (Fig. 4a) was conducted on genes and samples using centered correlation as the similarity metric and average linkage as the clustering method. Figure 4b shows the hierarchical clustering conducted for ncRNAs as described above for gene expression. Each color-bar unit in both hierarchical clustering represents a difference of one log 2 unit in RPKM value. Zero (0) is the median RPKM value across all samples. Heat maps with row dendograms are shown to visualize the result of the hierarchical clustering calculation. This depicts the distance or similarity between rows and which nodes in each row belongs to, as a result of clustering. For both the brain and spleen, the results of the clustering heat map show groups of genes or ncRNAs whose expression is affected by TBI and the exosome treatments. Principal component analysis showed similar distinction between groups (data not shown).

### RNA Seq of the brain and spleen transcriptome identifies genes affected by TBI and treatment with exosomes

We further analyzed the highly enriched genes for the spleen and brain. The results (above Fig. 4a, b) indicated specific sets of genes that were affected by TBI that were distinct between the spleen and brain as expected based on cell type and cell-specific markers. Based on fold changes, the top genes affected by TBI and treatment with exosomes in the spleen were enzymes such as prancreatic α-amylase, prancreatic α-amylase precursor, phospholipase A2, caroxypeptidase B precursor, bile salt-activated lipase precursor, chymotrypsinogen B precursor, carboxypeptidase A2 precursor, and anionic trypsin 1 which were elevated after TBI and reduced with exosome treatment. Further, in the spleen, genes that were upregulated following TBI were related to GO categories of enzyme inhibitor activity, defense response, platelet alpha granule lumen, and protein-DNA complexes. In the brain, our RNA Seq data revealed previously undescribed transcripts ENSRNOG00000047520, ENSRNOG00000049727, ENSRNOG00000050024, and ENSRNOG00000046742 whose levels changed with TBI and exosome treatment. Additionally, genes in the brain that were upregulated following TBI were related to immune responses, cellular response to stress, aging, and DNA damage. The splicing index of genes in the spleen that were predominantly changed with TBI and exosome treatment were *Tbcd*, *Chmp1a*, *Cnst*, *Lphn1*, *Zfp560*, *Mrpl23*, *Emc4*, *Zfp329*. The splicing index of genes in the brain that were predominantly changed with TBI and exosomes treatment were *Rps6ka3*, *Nrtk3*, *Nap1l1*, *Eif4g1*, *Fsd1*, *Trim44*, *Dst*, *Rps15*, *Fkbp2*, *Rabgap1l*.

### Gene expression patterns following treatment with hASC exosomes

Of most interest to this study are gene changes that fit the pattern observed with both behavioral testing and lesion area analysis. This expression pattern changes shown following TBI are lessened with exosome treatment but not by treatment with exosomes depleted of MALAT1. The genes that fit this pattern of gene expression responses were labeled (+ 1; up with TBI (T), down with TBI + exosomes (TE), and up with TBI + MALAT-depleted exosomes (TEdM)) or the opposite pattern (− 1; down with TBI (T), up with TBI+ exosomes (TE), and down with TBI + MALAT-depleted exosomes (TEdM)). The data was further filtered for a minimum of twofold gene expression change compared with TBI and analyzed using Ingenuity Pathway Analysis (IPA) for both the brain and the spleen separately. The IPA bioinformatics package identifies regulatory nodes (upstream regulators) based upon the fold change values of dataset to predict a relationship of these nodes to drive the detected changes in gene expression. In this manner, the top upstream regulators that fit the pattern + 1 and − 1 are listed for the brain and spleen in Table 1. Only values with a significant -log (*p* value) were included in the tables and if the Z score indicates a predicted significant up- or downregulation within each pattern than it is also noted in the table. Canonical pathways identified as significant are listed in Table 2.

**Table 1:**
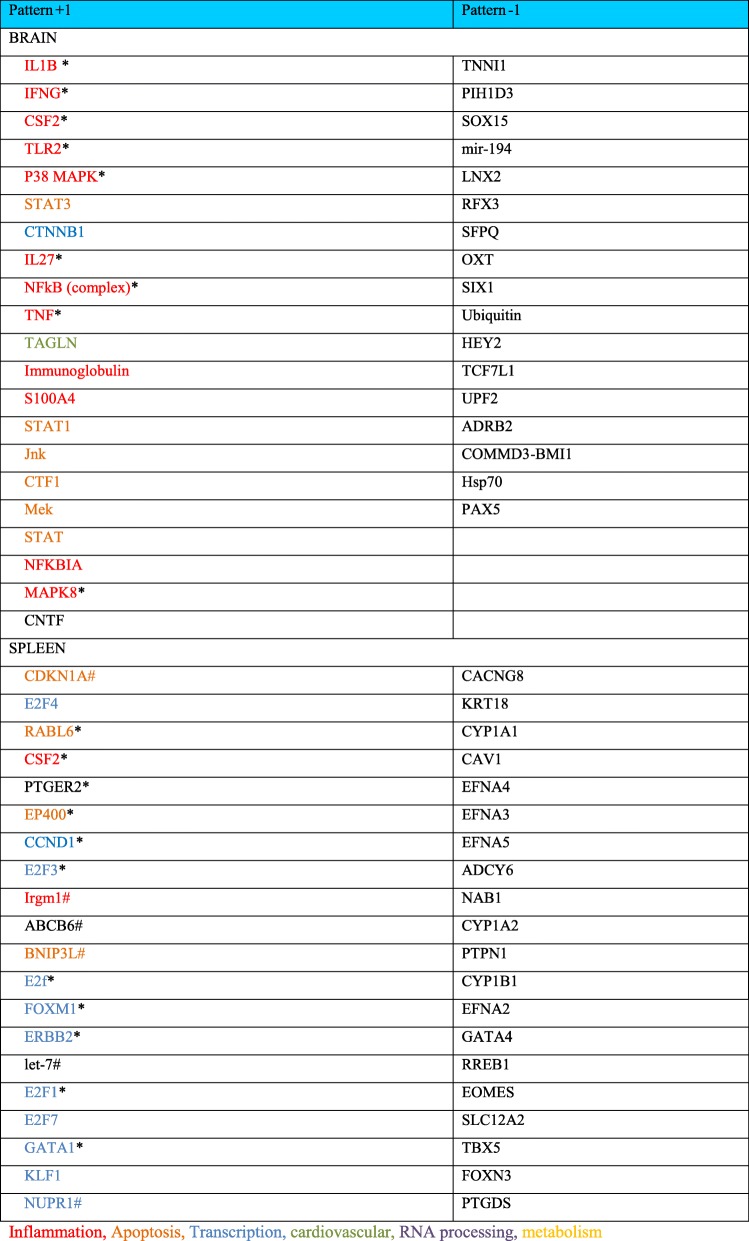
Upstream regulators

Asterisk indicates a positive *Z* score indicating a predicted activation

Number sign indicates a negative *Z* score

**Table 2:** Canonical pathways

Brain	
Pattern 1	
Pancreatic adenocarcinoma signaling	
Role of CHK proteins in cell cycle checkpoint control	
T helper cell differentiation	
Cyclins and cell cycle regulation	
Th1 pathway	
Bladder cancer signaling	
Inhibition of angiogenesis by TSP1	
Dopamine receptor signaling	
ILK signaling	
B cell development	
Cell cycle regulation by BTG family proteins	
Inhibition of matrix metalloproteases	
Thyroid cancer signaling	
HIF1α signaling	
Crosstalk between dendritic cells and natural killer cells	
Th1 and Th2 activation pathway	
Role of JAK family kinases in IL-6-type cytokine signaling	
Role of JAK1 and JAK3 in γc cytokine signaling	
Role of Oct4 in mammalian embryonic stem cell pluripotency	
Autoimmune thyroid disease signaling	
Spleen	
Pattern 1	Pattern − 1
Heme biosynthesis II	cAMP-mediated signaling
Mitotic roles of polo-like kinase	G-Protein coupled receptor signaling
Heme biosynthesis from uroporphyrinogen-III I	Serine biosynthesis
Tetrapyrrole biosynthesis II	Superpathway of serine and glycine biosynthesis I
Estrogen-mediated S-phase entry	LPS/IL-1-mediated inhibition of RXR function
GADD45 signaling	Protein kinase A signaling
Cell cycle: G2/M DNA damage checkpoint regulation	Cardiac hypertrophy signaling
Cyclins and cell cycle regulation	Fatty acid activation
dTMP de novo biosynthesis	nNOS signaling in skeletal muscle cells
Cell cycle control of chromosomal replication	Corticotropin releasing hormone signaling
DNA damage-induced 14-3-3σ signaling	
Rapoport-Luebering glycolytic shunt	
Pyrimidine deoxyribonucleotides de novo biosynthesis I	
ATM signaling	
Antiproliferative role of TOB in T cell signaling	
Role of CHK proteins in cell cycle checkpoint control	
Cell cycle: G1/S checkpoint regulation	
Cell cycle regulation by BTG family proteins	
Asparagine biosynthesis I	
γ-Glutamyl cycle	

#### Brain mRNA changes

**Fig. 5:**
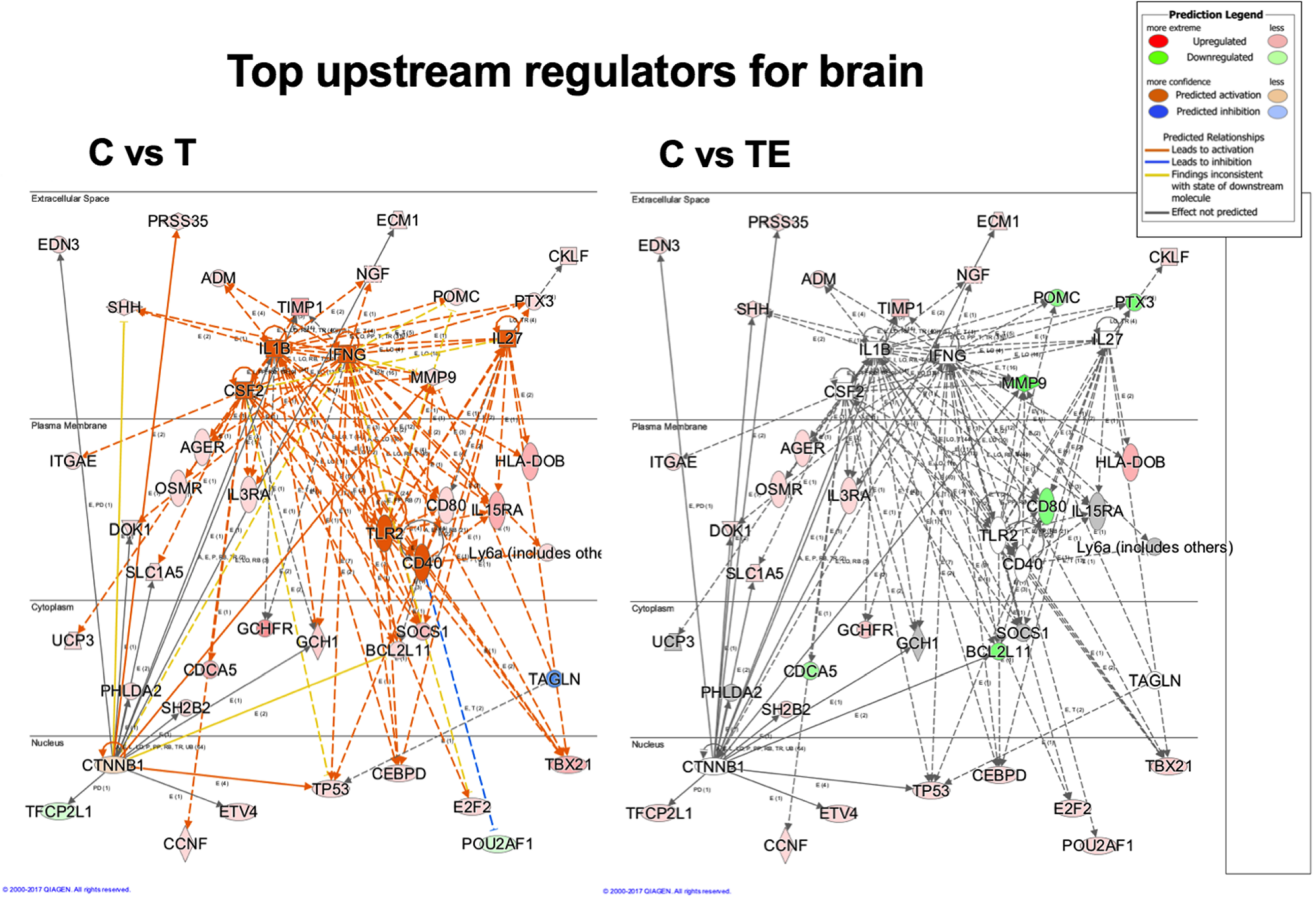
Upstream nodal regulators for brain. This panel illustrates several of the top predicted upstream nodal regulators for brain from the IPA analysis of the genes for the + 1 pattern. On the left C vs T, the upstream regulators IL1β, IFNG, IL27, CSF2, TLR2, CD40, and CTNNB1 are illustrated along with the gene expression driving their predicted upregulation. As you can see, there is significant overlap in the gene expression driving these upstream regulators. The gene expression patterning is shown with cellular location; thus, you can see the relationship between the nuclear transcription factors that are driving expression of targets that impact inflammation and cellular proliferation. On the right side of the panel, C vs TE, is an illustration of how gene expression in these networks changes with exosome treatment. All of the inflammation-related regulators move from a predicted activation depicted by the orange color on the left panel to no change from control as indicated by the lack of color in the right panel. A number of the genes driving these changes such as MMP9, TIMP1, POMC, and PTX3 which show upregulation following TBI (red) show downregulation (green) following treatment with exosomes. This shows a powerful modulation of many of the gene expression patterns following exosome treatment. Not shown in this figure is the pattern for treatment with exosomes depleted of MALAT1 as this gene expression network is almost identical to that observed on the left with TBI treatment alone. The legend inserted on the top right describes the meaning of the various color assignments

For the pattern + 1, several of the predominant predicted upstream nodal regulators from the IPA analysis are related to inflammatory pathways, IL1β, IFNG, etc. which is in agreement with the literature on TBI alone. To illustrate this graphically, Fig. 5 shows top upstream regulatory networks in the C vs. T comparison. There is a strong predicted upstream activation of inflammatory regulators such as IL1β, IFNG, TLR2, IL27, and CD40 (indicated in orange). Also illustrated in this figure are the actual gene expression changes driving the predictions, which show significant overlap in the nodal networks depicted. Furthermore, CTF1 was also predicted to be an upstream regulator by this analysis, which is consistent with its role in other brain injury models and inflammation. In addition, there were changes in CTNNB1: the gene for beta catenin which is an important regulator for the WNT pathway, WNT is known to be involved in neural repair pathways and neurogenesis, and is also related to SOX15 which is discussed further below. This pathway is downregulated with TBI, and the extent of down regulation is lessened following treatment with exosomes as reflected in the color of the regulator moving from a darker orange to a lighter color. The right side of the figure illustrates the overlay of the pattern of gene expression and predictions for the nodal regulation in the TBI treated with exosomes group. All of the inflammatory nodes such as IL1ß, IFNG, CD40, and IL27 show no predicted upregulation from control which is indicated by the lack of color, and demonstrates that the exosomes significantly lower the pro-inflammatory gene expression patterns observed with injury. This is driven by decreased gene expression of pro-inflammatory genes PTX3, MMP9, POMC, and CD80 in this dataset which is shown by the green color. Not shown in this figure is the network of these upstream regulators for the third aspect of this pattern which is C vs. TdM comparison; when exosomes depleted in MALAT1 are given after TBI, the network of gene expression and predicted upstream regulators is an almost identical pattern to the C vs. T network shown here. This is in agreement with the pattern of behavioral and histological changes presented above.

The predicted upstream regulators for the pattern − 1 with (gene expression going down with TBI) are related to transcription regulators such as SOX15, RFX3, and TCF7L1 as well as ubiquitin and HSP70 (Table 1, different functional groups depicted by colors). This suggests that there is a decline in transcriptional activation in regenerative pathways as illustrated by changes in SOX15 expression, which is part of the WNT/β-catenin pathway involved in cellular proliferation and stem cell proliferation.

For canonical pathways identified again the + 1 pattern shows the highest *p* value of overlap with no significant canonical pathways identified in the brain for the − 1 pattern. Canonical pathways that are increased with TBI and responsive to the exosome treatment also include inflammatory pathways. Cell cycle and cancer-related pathways are further noted, most likely changing due to cell death and injury caused by CCI (Table 2).

#### Spleen mRNA expression

We also examined gene expression in the spleen as our data shows predominant localization of exosomes to the spleen. This finding was important, as a significant body of literature has demonstrated that intravenous treatments with exogenous cell therapies and other treatments such as conditioned media from stem cells involve the spleen. Our data presented here shows the presence of exosomes in the spleen at an early time point following injection, prior to the exosomes being observed in the brain. Table 1 shows the top ranking predicted upstream regulators based upon *p* value of overlap, and if there was a significant *Z* score for predicted activation or inhibition. For the + 1 pattern of expression that increases with TBI, there was a predicted inhibition of CDKN1A suggesting a decrease in cellular proliferation. This is supported by changes in other proliferation-related pathways like CCND1, which is a cell cycle regulator related to both AMPK and PTEN bioenergetics pathways as well as inflammation pathways like IL8 and ILK. Many of the predicted upstream regulators are nuclear transcription factors influencing many aspects of cellular function including cellular proliferation, cell cycle control, and DNA damage. This again is likely a reflection of the response to the brain injury effecting other organ systems.

**Fig. 6:**
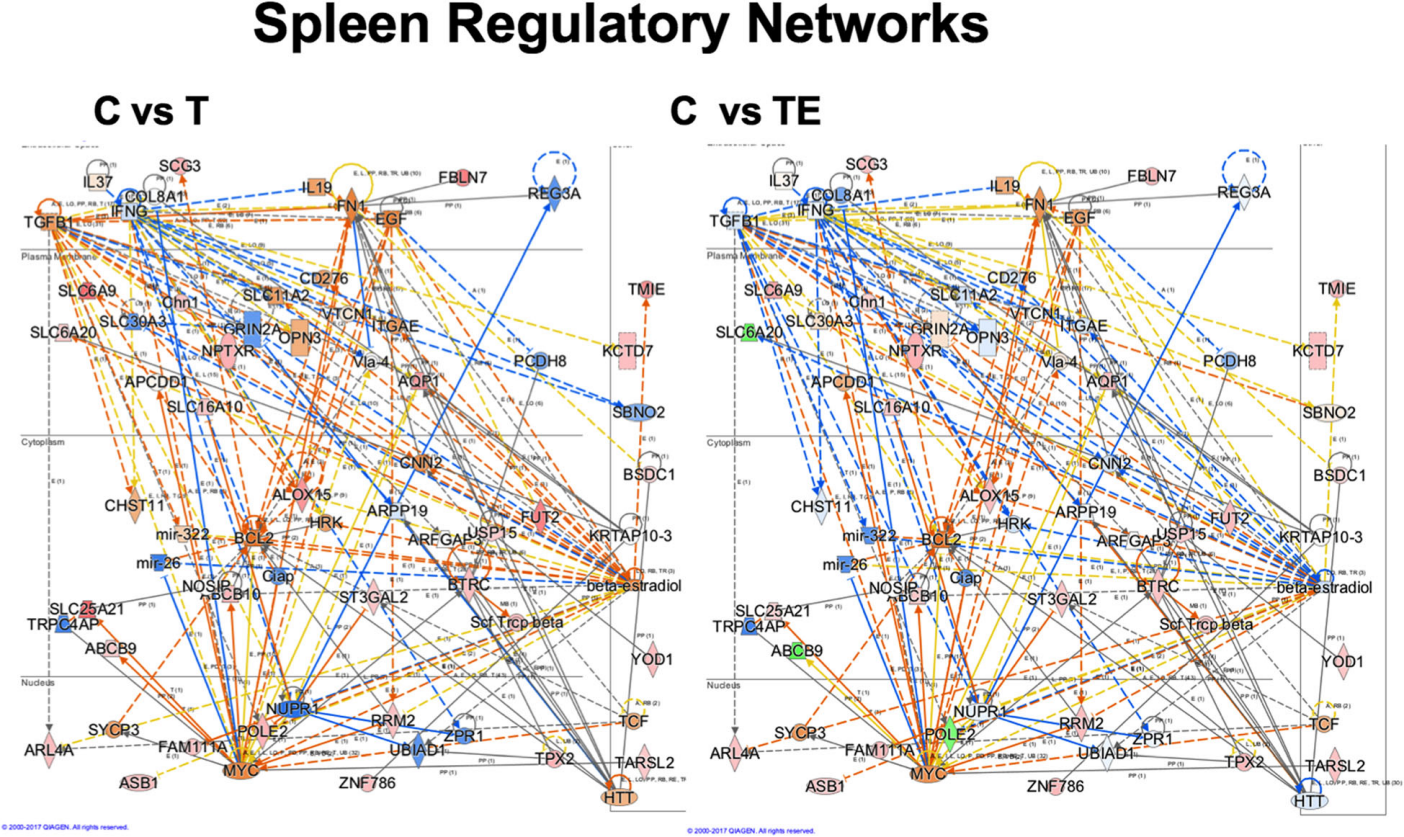
Spleen regulatory networks. Gene networks altered by TBI in the spleen are depicted in this figure. On the left C vs T is the gene expression in these networks when comparing the control and TBI gene expression showing activation of inflammatory pathways TGFB1, IL19, and IL37. BCL2 is activated, likely in response to the impact of the brain injury as a compensatory process in the spleen. NUPR1 is a transcriptional cofactor involved in apoptosis and is reciprocally inhibited by TBI. In the right panel C vs TE, it is shown that exosome treatment returns NUPR1 expression back towards control levels. Also of interest is a predicted regulation of these networks by beta-estradiol activation following TBI, which is inhibited following exosome treatment. A number of genes related to this pathway are the inflammatory changes and also mir-322 which is responsive to hypoxia and oxidative stress [32]. Beta-estradiol is known to play a role in splenic response to injury by modulating macrophage and dendritic cell function

To explore the regulatory networks of genes affected in the spleen in response to the + 1 pattern of expression, we merged two of the top networks identified in IPA and this is illustrated in Fig. 6. The left panel shows these network changes along with predicted nodes in addition to relationships with canonical pathways. Again, as in the brain, a number of inflammation-related pathways including TGFβ and INFG are altered with TBI. Also shown are a number of changes to pathways related to the transcriptional regulators MYC and NUPPR1. The predicted inhibition of NUPPR1 with TBI is mitigated with the exosome treatment as shown in the right panel and might be related to an increase in POLE2 in the exosome-treated group. Also of interest is a predicted regulation of these networks by beta-estradiol activation following TBI which is inhibited following exosome treatment (Fig. 6). A number of genes related to this pathway are involved in inflammatory responses and cellular stress, such as mir-322 which is responsive to hypoxia and oxidative stress [32]. Beta-estradiol is known to play a role in splenic response to injury [33–36] by modulating macrophage and dendritic cell function, thus are consistent with effects of estradiol in other injury models. It is important to note that this does not necessarily mean that estradiol is driving this alteration in our dataset; it simply means that estradiol alters splenic inflammatory markers in the same way as what is observed in our dataset. Several investigations have suggested that estradiol reduces MALAT1 [37–39]; thus, a predicted inhibition of estradiol by exosome treatment may reflect increased action of MALAT1. Further studies are needed to elucidate an interaction of exosome treatment with estradiol.

The expression of genes in the − 1 pattern demonstrated inhibition of several aspects of splenic function including the ephrins A2, 3, 4, and 5, which regulate interaction with hematopoiesis [40]. While these processes are more related to ephrin A1, for our study, ephrin A2 is of particular interest as it plays an important role in monocyte adhesion and transendothelial migration via integrins. In a report by Konda et al., an increase in ephrin A2 stimulated monocyte infiltration and sequestration into the red pulp of the spleen via an interaction with integrin and additional undetermined molecular pathways [41]. The release of monocytes from the red pulp of the spleen is a known consequence of injury [42]; therefore, we would predict that inhibition of ephrin A2 would be consistent with release of monocytes from the splenic red pulp into the bloodstream. As presented, this predicted loss of the inhibition of ephrins is seen following treatment with exosomes, but treatment with exosomes depleted of MALAT1 showed similar results to those observed for rats that received TBI with no treatment. This is a potentially interesting finding, and although at this point we cannot make a direct correlation between this predicted change in ephrin A2 regulation following TBI and treatment with exosomes, it is consistent with the observed behavioral and histological improvement with the exosome treatment. As previously discussed, one possible role of the spleen in TBI and other brain insults is the release of monocytes into circulation, which participate in the secondary insult at the site of injury.

### Validation of RNA Seq results of gene expression

**Fig. 7:**
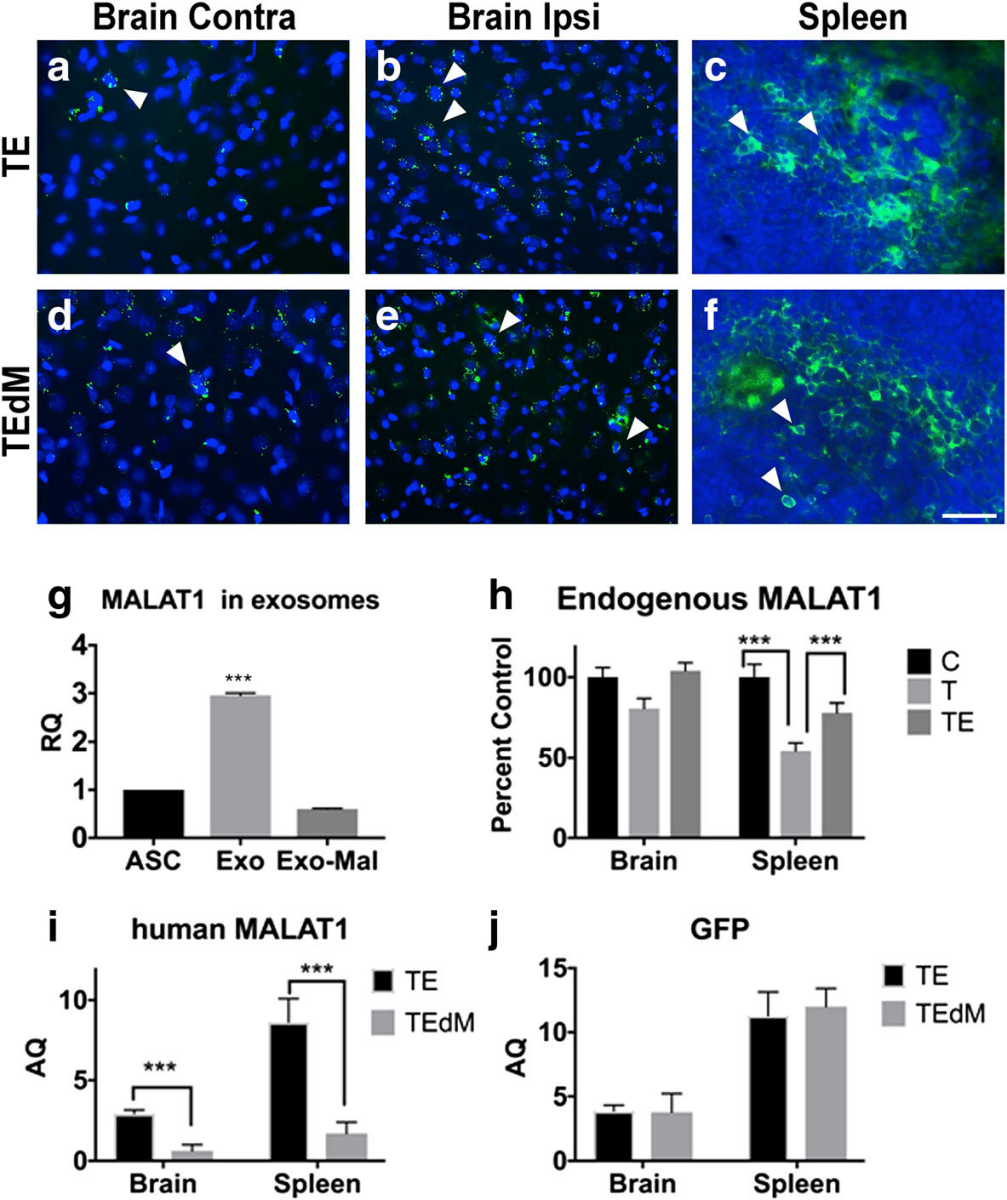
Localization of exosomes and validation of MALAT1 and GFP levels. Localization of exosomes and MALAT1 to the brain and spleen. Confocal imaging of exosome-positive expression of GFP in the brain and spleen of TBI rats. Images reveal migration of exosomes to both the contralateral and ipsilateral cortical region of the TBI-impacted brains near the impact site (**a**, **b**, **d**, **e**) and the spleen (**c**, **f**) of TE and TEdM rats as shown by detecting GFP expression (green) in cells with DAPI-positive staining (blue) 11 days after transplantation. White arrows indicate cells with exosomes. There was higher migration to the ipsilateral cortex near the impact site than that observed in the contralateral cortex which agrees with our ex vivo imaging data in Fig. 3. Scale bar in **f** equals 50 μm. **g** MALAT1 expression was measured in hASC-isolated exosomes and isolated exosomes with MALAT1 knockdown. Relative to the MALAT1 level in hASC, there is an increase in MALAT1 in the secreted exosomes, and this was knocked down significantly with the antisense oligonucleotide treatment. The results were analyzed with two-tailed Student’s *t* test using PRISM4 statistical analysis software (GraphPad, San Diego, CA). A level of *p* < 0.05 was considered statistically significant. ***p < 0.001. **h** Endogenous MALAT1 levels in rat brain and spleen decrease with TBI: Following TBI and treatment with hASC exosomes, the levels of MALAT1 within the rat brain and spleen were analyzed by PCR using rat-specific primers. RNA was extracted and samples were pooled from four rats each from the no TBI, TBI treated with vehicle, and TBI treated with exosomes. Graph represents percent control with no TBI group designated as 100%. The experiment was repeated five times. The results were analyzed with two-tailed Student’s *t* test using PRISM4 statistical analysis software (GraphPad, San Diego, CA). A level of *p* < 0.05 was considered statistically significant. ***p < 0.001 highly significant between control (no TBI) and TBI with vehicle treatment and between TBI and TBI treated with hASC exosomes for MALAT1. **i** Verification that hASC exosomes were taken up by the brain and spleen: SYBR Green real-time qPCR using human MALAT1 primers and **j** GFP primers was performed for absolute quantification. GAPDH served as control. For absolute quantification, a standard curve was generated for each gene in every assay. Absolute quantification of mRNA expression levels for MALAT1 and GFP was calculated by normalizing the values to GAPDH. The experiments were repeated four times with similar results. The results were analyzed with two-tailed Student’s *t* test using PRISM4 statistical analysis software (GraphPad, San Diego, CA). A level of *p* < 0.05 was considered statistically significant. ***p < 0.0001 highly significant between TBI treated with hASC exosomes and TBI treated with MALAT1-depleted (M1 aso) exosomes for MALAT1. The levels of GFP did not change with depletion of MALAT1

Confocal microscopy confirmed positive localization of GFP-labeled exosomes within both the contralateral and ipsilateral brain regions as well as in the spleen (Fig. 7a–f). The GFP signal was more robust in the ipsilateral hemisphere compared to the contralateral and was located mainly in close proximity to the impacted area. This may be the result of a breakdown of the blood-brain barrier at the site of injury. To further validate that exosomes were taken up by the brain and spleen, RNA isolated from either the brain or the spleen was reverse transcribed to cDNA and used in quantitative SYBR Green real-time qPCR using primers for rat and human MALAT1. Our results show that endogenous rat MALAT1 is depleted in the spleen and the cortex near the injury site following injury and increased with exosome treatment. Further, exosomes not depleted of MALAT1 were shown to deliver human MALAT1 to the brain and spleen (Fig.7g, h). These results show that exosomes are efficiently taken by the brain and spleen.

**Fig. 8:**
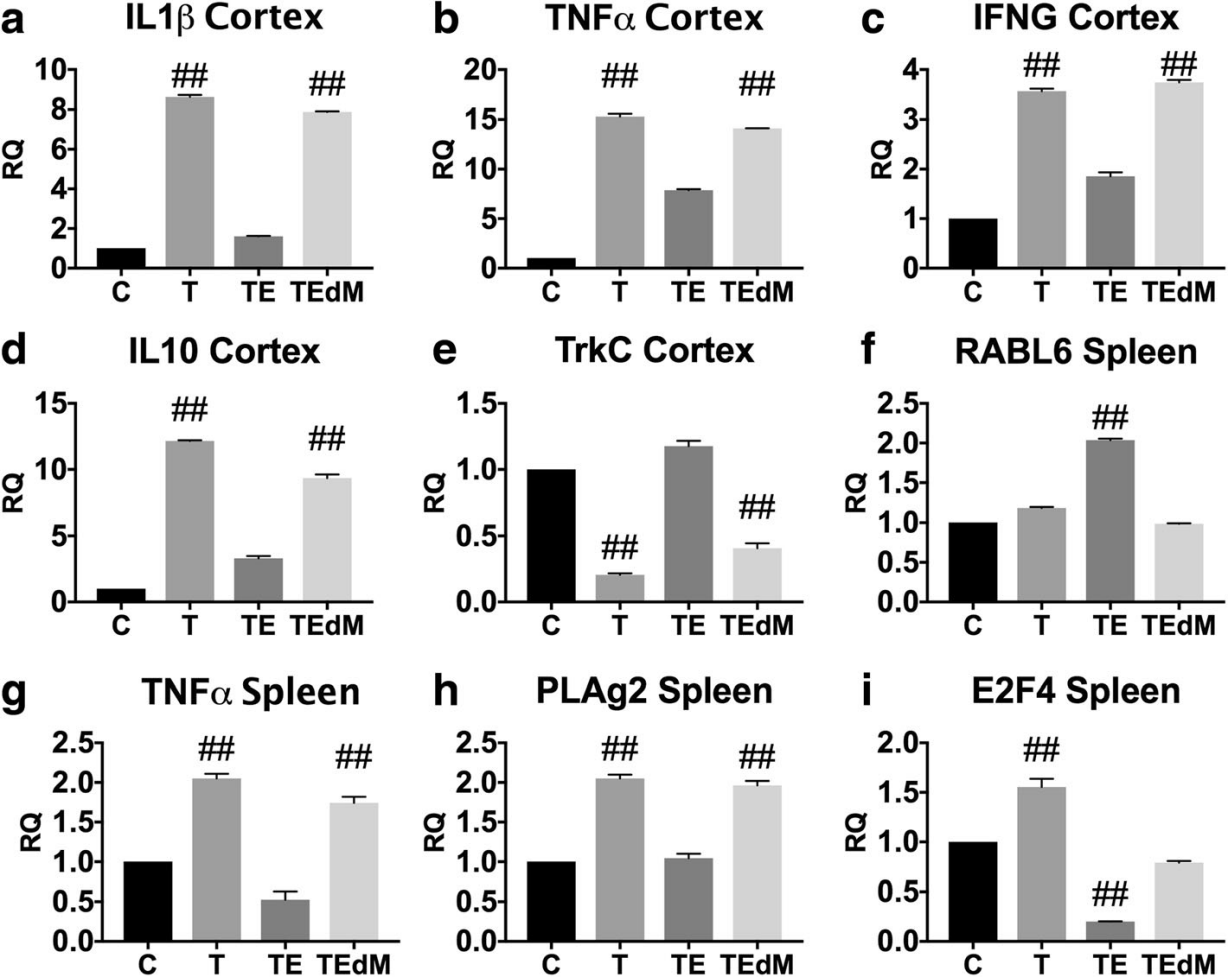
**a**–**i** Validation of RNA Seq data using qPCR. Real-time qPCR validation of representative genes identified by RNA Seq data in the cortex and spleen that are affected by TBI and treatment with exosomes. All assays were run in triplicate, and data is expressed as the mean ± SEM. Data for each gene was analyzed by one-way ANOVA followed by Dunnet’s multiple comparison test. ^##^*p* < 0.01 indicates that group was different from control. ANOVA for each graph are IL1ß cortex (*F*(3, 8) = 16,008); TNFα cortex (*F*(3, 8) = 5989); IFNG cortex (*F*(3, 8) = 1784); TrkC cortex (*F*(3, 8) = 814); IL10 cortex (*F*(3, 8) = 2980); RABL6 spleen (*F*(3, 8) = 4640); TNFα spleen (*F*(3, 8) = 286); E2F4 spleen (*F*(3, 8) = 516); PLAg2 spleen (*F*(3, 8) = 451)

To validate our results from RNA Seq, we used quantitative SYBR Green real-time qPCR to determine levels of the genes altered in both the spleen and the brain following TBI with and without exosome treatment. Figure 8 shows validation of spleen expression of PLAg2, TNFα, E2F4, and RABL6 and brain cortex expression of IL1β, TNFa, IFNG, and IL10. These genes showed significant fold changes in RNA Seq data and are components of the predicted networks. Although the pattern of expression for IL10 is not consistent with the overall predicted changes in inflammatory networks, it does replicate the data in the RNA Seq dataset. Our qPCR results show expression of these genes that are in agreement with the RNA Seq data.

### Noncoding RNA levels change with injury and exosome treatment in a MALAT1-dependent manner

Long noncoding RNAs promote epigenetic modifications and regulate gene expression by direct interaction with pre-mRNA of genes or by acting as scaffolding to tether a complex of micro RNAs to promote transcription of genes. There is an emerging appreciation for its ability to not only interact with other noncoding RNAs but also promote transcription of these noncoding RNAs. Among the noncoding RNAs (ncRNAs) detected in the brain and spleen, a large number are associated with tRNA, rRNA, and small nuclear RNAs (snRNAs) whose function is constitutive in transcription and translation processes. Our study points to two primary classes of ncRNAs that are directly affected by the exosomes containing MALAT1: the microRNAs (miR) and the small nucleolar RNAs (snoRNAs).

miRNAs regulate gene expression by directly binding to their target mRNA. Our RNA Seq data identified miR-200b, miR-200a, miR-183, miR-182, miR-96, and miR-451a as being significantly upregulated four- to eightfold in the brain with TBI (Table 3). These miRNAs were previously shown to regulate apoptosis in various diseases [43–52]. Significantly, treatment with exosomes downregulated their expression; however, MALAT1-depleted exosomes did not have a significant effect on the expression of these miRNAs. When the RNA Seq data of the brain is sorted and analyzed by the + 1 pattern described above, we note that miR-96-5p, miR-182, miR-183-5p, miR-200b-5p, miR-429, mir-6216, miR-1193-3p, and miR-218a-2-3p increase following TBI, decreased by exosome treatment, and the reversal is not seen with MALAT1-deficient exosomes. miR200b-3p and miR-182-5p are increased following TBI in the brain, the latter of which is directly modulated by MALAT1. Both of these miRNAs also show a reduction following treatment with exosomes, but not exosomes depleted of MALAT1, opening the possibility that MALAT1 in exosomes may be responsible for the shift in pattern. A second set of miRNAs include miR-487b-3p, miR-672-3p, miR-10a-5p, miR-770-5p, miR-666-5p, miR-29c-3p, miR-139-3p, miR-668, miR-344a-3p, and miR1843a-3p that follow the − 1 pattern such that they decrease following TBI, increased by exosome treatment, and the reversal is not seen with MALAT1-deficient exosomes. Of the miRNAs whose expression changes with injury and exosome treatment, miR10a-5p follows the − 1 pattern in the brain and the spleen. miR10a-5p is extensively studied for its role in the development and regulation of TOP mRNAs [53]. miR10a-5p promotes expression of ribosomal proteins, elongation factors, and other proteins associated with translation apparatus, thereby influencing global protein synthesis. miR10a-5p expression is increased in stem cells and in our experiments, making it possible that miR10a-5p is part of the exosomal cargo, which is transferred both to the brain and spleen, and has a predominant role in enhancing the response to injury and aiding the recovery upon treatment with exosomes.

**Table 3:** Changes in noncoding RNA miRNA

Brain + 1						Spleen + 1			
		C vs T	C vs TE	C vs TEdM			C vs T	C vs TE	C vs TEdM
rno-miR-429		346.52	26.50	208.76		rno-miR-451-3p	3.70	− 1.15	4.82
rno-miR-200b-5p		99.01	7.42	127.07		rno-miR-1949	1.78	− 1.05	1.45
rno-miR-183-5p		59.14	6.56	122.37		rno-miR-6216	1.38	− 1.22	1.30
rno-miR-182		56.38	1.00	43.57		rno-miR-17-5p	1.51	− 1.13	1.52
rno-miR-96-5p		48.13	1.00	61.72		rno-miR-144-5p	3.85	2.01	4.19
rno-miR-6216		2.67	− 1.04	2.82		rno-miR-484	1.52	− 1.10	1.88
rno-miR-1193-3p		1.34	1.03	1.20		rno-miR-132-3p	2.07	1.01	1.83
rno-miR-218a-2-3p		1.24	1.07	1.30		rno-miR-702-3p	4.89	2.44	3.59
						rno-miR-409a-5p	1.85	1.04	1.84
						rno-miR-92a-3p	1.33	− 1.08	1.29
						rno-miR-339-5p	1.39	− 1.07	1.30
						rno-miR-93-5p	1.53	1.07	1.74
						rno-miR-361-5p	1.30	− 1.01	1.50
						rno-miR-31a-5p	1.18	− 1.13	1.30
						rno-miR-21-3p	1.29	− 1.07	1.31
						rno-miR-423-5p	1.19	− 1.10	1.26
						rno-miR-210-3p	1.35	1.11	1.45
						rno-miR-532-3p	1.25	1.11	1.26
						rno-miR-103-3p	1.18	− 1.01	1.24
						rno-miR-6319	1.39	1.12	1.27
						rno-miR-378a-5p	1.10	1.05	1.17
						rno-miR-342-3p	1.06	− 1.05	1.09
Brain −1						Spleen −1			
rno-miR-154-5p		− 1.48	−1.17	− 1.32		rno-miR-708-3p	− 10.9	1.69	− 1.39
rno-miR-7b		− 1.65	−1.04	− 1.80		rno-miR-6329	− 3.33	− 1.07	− 1.57
rno-miR-665		− 2.57	−1.11	− 1.69		rno-miR-148b-5p	− 1.22	1.14	− 1.10
rno-miR-487b-3p		− 1.38	1.32	− 1.54		rno-miR-212-5p	− 1.44	− 1.09	− 2.17
rno-miR-672-3p		− 1.77	1.09	− 1.41		rno-miR-181a-1-3p	− 1.55	1.23	− 1.16
rno-miR-10a-5p		− 1.47	1.29	− 1.29		rno-miR-1843b-5p	− 1.79	1.07	− 1.37
rno-miR-770-5p		− 1.53	1.12	− 1.60		rno-miR-10a-5p	− 1.49	1.16	− 1.22
rno-miR-666-5p		− 1.29	1.24	− 1.32		rno-miR-379-5p	− 1.92	− 1.01	− 1.32
rno-miR-29c-3p		− 1.52	1.07	− 1.22		rno-miR-411-5p	− 1.66	− 1.12	− 2.09
rno-miR-3594-3p		− 1.61	− 1.04	− 1.37		rno-miR-192-5p	− 1.72	1.01	− 1.80
rno-miR-485-3p		− 1.41	− 1.13	− 1.32		rno-let-7f-5p	− 1.58	1.12	− 1.20
rno-miR-139-3p		− 1.45	1.02	− 1.27		rno-miR-1b	− 1.53	1.14	− 1.37
rno-miR-139-5p		− 1.54	− 1.09	− 1.41		rno-miR-148a-3p	− 1.40	− 1.10	− 1.20
rno-miR-668		− 1.19	1.12	− 1.20		rno-miR-126a-5p	− 1.46	1.09	− 1.47
rno-miR-342-5p		− 1.15	1.10	− 1.11		rno-miR-28-5p	− 1.39	1.08	− 1.30
rno-miR-344a-3p		− 1.18	1.01	− 1.21		rno-miR-204-5p	− 1.47	1.00	− 1.20
rno-miR-330-3p		− 1.30	− 1.05	− 1.19		rno-miR-181c-3p	− 1.67	− 1.14	− 1.42
rno-miR-1843a-3p		− 1.13	1.01	− 1.16		rno-miR-3068-3p	− 1.58	− 1.08	− 1.24
rno-miR-328a-3p		− 1.18	− 1.08	− 1.18		rno-miR-30c-5p	− 1.41	1.03	− 1.15
						rno-miR-27a-3p	− 1.16	1.06	− 1.21
						rno-miR-139-5p	− 1.33	− 1.07	− 1.24
						rno-miR-140-5p	− 1.30	1.06	− 1.25
						rno-miR-337-5p	− 1.42	− 1.05	− 1.34
						rno-miR-30a-5p	− 1.16	1.03	− 1.08
						rno-miR-150-5p	− 1.29	− 1.10	− 1.16
						rno-miR-147	− 1.12	1.04	− 1.09
						rno-miR-30e-5p	− 1.24	− 1.08	− 1.14
ncRNA including snoRNA, snRNA									
Brain + 1					Brain − 1				
		C vsT	C vs TE	C vs TEdM			C vsT	C vs TE	C vs TEdM
snRNA	U6	1.57	1.00	1.37	snoRNA	SNORD42	− 1.90	− 1.24	− 1.82
snoRNA	SNORD24	1.28	1.11	1.38	lncRNA	PVT1 3	− 1.98	− 1.34	− 2.18
snoRNA	SNORD60	1.25	1.00	1.22	snoRNA	SNORA28	− 1.29	− 1.07	− 1.97
snRNA	U6	1.09	1.00	1.06	rRNA	Rnr2	− 1.25	− 1.07	− 1.23
snoRNA	ACEA U3	1.64	1.05	1.59	rRNA	Rnr2	− 1.09	− 1.10	− 1.29
snoRNA	SNORA62	1.51	− 1.04	1.27	lncRNA	RMST 2	− 1.19	− 1.06	− 1.13
snoRNA	SNORA73	1.39	− 1.24	1.30					
snoRNA	SNORD62	1.76	− 1.21	1.32					
snoRNA	SNORD15	3.17	1.38	3.12					
snoRNA	SNORA38	1.06	− 1.00	1.06					
Spleen + 1					Spleen − 1				
snoRNA	SNORD74	1.85	− 1.39	1.52	snoRNA	SNORA41	− 3.84	− 1.14	− 1.85
snRNA	U6	1.67	− 1.27	1.67	snoRNA	SNORD33	− 3.68	− 1.64	− 3.91
snoRNA	SNORD75	1.68	− 1.03	1.50	snoRNA	SNORA54	− 3.49	1.06	− 1.98
scRNA		1.32	− 1.23	1.14	snoRNA	SNORD21	− 3.48	− 1.48	− 3.88
lncRNA	LOC100909539	1.37	− 1.06	1.48	snoRNA	SNORA71	− 2.94	− 1.55	− 3.67
misc_RNA		1.31	1.10	1.42	vault_RNA	Vault	− 2.93	− 1.49	− 5.34
lncRNA	Bc1	1.15	1.04	1.25	snoRNA	SNORA11	− 2.71	− 1.08	− 2.24

SnoRNAs function as sequence-specific guides to direct site-specific nucleoside modifications for other noncoding RNAs such as rRNA and snRNA. A select few snoRNAs are shown to interact with mRNA and regulate its splicing [54–56]. Recent breakthroughs have predicted the interaction of lncRNAs with snoRNAs [57]; however, their association are not shown in vivo. SnoRNAs are transcribed as nuclease products from genes. Our data shows, for the first time, that a long noncoding RNA MALAT1 regulates the expression of snoRNAs in the brain. Notably, SNORA31, SNORD33, SNORD64, SNORA18, SNORA17, SNORD44, SNORD47, SNORD28, SNORD113, SNORD62, SNORA29, and SNORD2 were upregulated two- to fourfold with exosome treatment following TBI; MALAT1-depleted exosomes did not increase the levels of these snoRNAs (Table 3). Brain-specific snoRNAs in mice MBI48 and MBI52 were shown to promote memory and learning [58, 59]. While lncRNAs are known to have a role in pre-mRNA splicing, mRNA editing, and mRNA stability control, this is the first evidence showing that lncRNA MALAT1 regulates the expression and transcription of brain snoRNAs.

## Discussion

In summary, we have demonstrated that exosomes secreted from hASCs have a beneficial role in modulating pathology following mild TBI, particularly those containing MALAT1. Our results have shown an attenuation in TBI-induced motor deficits as well as decreased cortical damage at both the impact and peri-impact level after administration of exosome treatment that is dependent on MALAT1. Our previous in vitro study using HT22 cells demonstrated that MALAT1 has a role in neuroprotection in vitro. Indeed, we showed that hASC-derived exosomes increase neuronal survival and proliferation in vitro in the context of several models of injury and this activity is lost when the exosomes are depleted of MALAT1 [27]. These results suggest that intravenous administration of exosomes generated from hASCs may represent a novel therapeutic approach for treatment of TBI. This is consistent with the report that MALAT1 regulates pathology following ischemic injuries [28]. To distinguish the actions of subcomponents of conditioned media, we treated rats with CM that did not contain exosomes and found it had a reduced effect on most measures when compared to exosome treatment. However, there was still some effect seen from CM depleted of exosomes. The difference between the exosome treatment and the CM depleted of exosomes suggests the cargo found within the exosome is important for providing significant neuroprotection and promoting regeneration after injury. Secondly, the effect shown with CM treatment, even depleted of exosomes, suggests the possibility that the CM maintains valuable secreted factors.

Exosomes are known to hold a plethora of biological molecules in its complex cargo including proteins, lipids, mRNAs, and miRNAs capable of interacting with surrounding cells in order to modify the host environment [60]. Exosomes generated from MSCs have been shown to significantly increase brain angiogenesis and neurogenesis as well as reduce neuroinflammation; however, the specific molecular constituents driving regeneration were not identified in most studies [20]. It is thought that RNA cargo plays an important role in the cell-to-cell communication driving neuroprotection, making it a key component in promoting the recovery seen following injury. Our study demonstrates that lncRNA MALAT1 is a critical component of the hASC exosomes and drives the recovery process. Many single molecular pathway approaches have shown little or no promise in clinical trials [61], and this emphasizes a need for combination therapies to tackle the complexity of secondary neurological injury post TBI. Treatment with exosomes is considered to be a multi-targeted approach as they are able to seek and modulate multiple targets and enforce a neuroprotective environment, providing further promise for their use as a therapeutic approach.

In this study, we demonstrate for the first time the in vivo and ex vivo biodistribution of intravenous DIR-labeled exosomes derived from hASCs. It is well known that when administering cell therapy intravenously, the cells migrate to multiple organs and here we show exosomes migrating to the same organ sites, with some differences in time course and degree of migration. Most importantly, we found that the exosomes are observed in the spleen and liver area within 1 h of IV treatment when observed both in vivo and ex vivo, followed by a decrease in radiance efficiencies over time. Though migration to the liver probably serves as a filtering and elimination process, the migration of the exosomes to the spleen implicates a probable immune interaction. It has been shown that injury induces the release of immune cells from the spleen with consequent infiltration into the brain [62] and that this is a significant aspect of the secondary insult following TBI as discussed in the introduction. It has been shown that blocking entry of inflammatory monocytes into the brain can significantly reduce the observed damage and offers recovery of cognitive function [9]. The spleen is one source of peripheral macrophages and monocytes, and it is possible that treatment with exosomes inhibits the release of these immune cells into circulation. This in turn lessens the infiltration into the brain through the disrupted BBB and prevents the contribution of peripheral immune cells to the secondary injury. This was supported by the observed inhibition of ephrin family gene expression following TBI as a predicted upstream regulator in the spleen following injury. This inhibition was modulated by exosome treatment but not by treatment with exosomes depleted of MALAT1. Ephrins are regulators of transendothelial migration of monocyte/macrophages and have been shown to play a role in monocyte adhesion in the red pulp of the spleen [41]. Thus, a decrease with TBI could be reflective of a migration of monocytes into the circulation. Therefore, the observed migration of the exosomes to the spleen immediately after administration could be an important aspect of the mechanism of action that elicits the improvement in both cellular and functional recovery as seen here post TBI. Future studies can look deeper into this potential mechanism of action and the role of MALAT1 at the level of the spleen.

Exosomes were also observed in the brain, albeit with a much lower degree of signal and with a different time course than that observed for the spleen. Exosomes were primarily observed around the site of injury. The BBB is known to be disrupted by TBI [63] peaking at 4–6 h and 2–3 days after injury [64] and therefore may explain one factor for the observed delayed localization of exosomes. Alternatively, it is also possible that there is expression of chemokine or signaling molecules that would attract the exosomes to the area of injury. At this time, we do not know if the primary site for the therapeutic effect of the exosomes is at the level of the spleen or the brain, and it is likely that both play some role. It has been shown that hASC-derived exosomes like those studied here have direct neuroprotective effect on neuronal cells [27] and this is another area of future investigation.

RNA Seq is a robust method to evaluate the effect of TBI at the genomic level. We have identified genes and pathways that are affected by TBI as well as the genomic influences of exosome treatment. Our study has demonstrated that treatment with exosomes post TBI significantly improves the outcome by modulating gene networks. The knowledge of specific genes and pathways as well as the influence of noncoding RNA in exosome treatment post TBI is crucial to develop an understanding of the multitude of events that occur simultaneously to promote healing. Our data indicates that not only genes involved in survival are affected but also those genes that are involved in the inflammatory and immune response to injury. The cargo carried by the exosomes is pivotal in cell-cell communication and influences the response to injury and recovery. Exosomes from hASC contain not only lncRNA MALAT1 but also additional lncRNAs and proteins [29]. Our study specifically focused on the role of MALAT1; however, as shown in the data, other ncRNAs or proteins carried in the exosomes may also aid in the recovery process. Further studies are being undertaken to delineate additional players and the complex interactions between lncRNAs and other RNA and proteins.

The above results demonstrate that the lncRNA MALAT1 regulates expression of mRNA and ncRNA involved in the inflammatory response, apoptosis and cell survival, signal transduction by MAPK pathways, and transcription of genes. There is also an emerging appreciation for the ability of lncRNA to not only interact with other ncRNAs but also promote transcription of these ncRNAs. Our data demonstrates for the first time that a lncRNA promotes expression of snoRNAs. Our dataset contains a tremendous amount of information on novel and known genes in the brain and spleen that were not previously recognized. This RNA Seq data provides a valuable tool to elucidate additional genes and ncRNAs in the brain and spleen and provides a robust biological system to validate its findings.

## Conclusions

Taken together, our data demonstrate that hASC-derived exosomes containing MALAT1 have a beneficial effect on function and pathology following a CCI injury. Importantly, we show that the lncRNA MALAT1 affects not only mRNA expression but also expression of noncoding RNA. Thus, the action of the exosomes is multifold and impacts a number of cell survival, inflammatory, and regulatory pathways in both the spleen and the brain. As exosomes show tremendous promise as therapeutics, understanding the importance of the cargo of the exosomes is critical for understanding their mechanism of action as well as determining how to identify the exosomes with the most potential for benefit.

## Abbreviations

BBB: Blood-brain barrier
CCI: Controlled cortical impact
CDC: Center for Disease Control
EBST: Elevated body swing
GFAP: Glial fibrillary acid protein
GFP: Green fluorescent protein
hASC: Human adipose-derived stem cell
lncRNA: Long noncoding RNA
MALAT1: Metastasis-associated lung adenocarcinoma transcript 1
miRNA: Micro RNA
mRNA: Messenger RNA
MSC: Mesenchymal stem cell
ncRNA: Noncoding RNA
RNA: Ribonucleic acid
SGZ: Subgranular zone
SVZ: Subventricular zone
TBI: Traumatic brain injury
